# Exosomes and Extracellular Vesicles as Emerging Theranostic Platforms in Cancer Research

**DOI:** 10.3390/cells9122569

**Published:** 2020-12-01

**Authors:** Giorgia Ailuno, Sara Baldassari, Francesco Lai, Tullio Florio, Gabriele Caviglioli

**Affiliations:** 1 Department of Pharmacy, University of Genova, 16147 Genova, Italy; ailuno@difar.unige.it (G.A.); baldassari@difar.unige.it (S.B.); 2 Department of Life and Environmental Sciences (DiSVA), University of Cagliari, 09124 Cagliari, Italy; frlai@unica.it; 3 Department of Internal Medicine, University of Genova, 16132 Genova, Italy; 4 IRCCS Ospedale Policlinico San Martino, 16132 Genova, Italy

**Keywords:** exosomes, extracellular vesicles, diagnosis, theranostic

## Abstract

Exosomes are endosome-derived nanovesicles produced by healthy as well as diseased cells. Their proteic, lipidic and nucleic acid composition is related to the cell of origin, and by vehiculating bioactive molecules they are involved in cell-to-cell signaling, both in healthy and pathologic conditions. Being nano-sized, non-toxic, biocompatible, scarcely immunogenic, and possessing targeting ability and organotropism, exosomes have been proposed as nanocarriers for their potential application in diagnosis and therapy. Among the different techniques exploited for exosome isolation, the sequential ultracentrifugation/ultrafiltration method seems to be the gold standard; alternatively, commercially available kits for exosome selective precipitation from cell culture media are frequently employed. To load a drug or a detectable agent into exosomes, endogenous or exogenous loading approaches have been developed, while surface engineering procedures, such as click chemistry, hydrophobic insertion and exosome display technology, allow for obtaining actively targeted exosomes. This review reports on diagnostic or theranostic platforms based on exosomes or exosome-mimetic vesicles, highlighting the diverse preparation, loading and surface modification methods applied, and the results achieved so far.

## 1. Introduction

### 1.1. Classification and Characteristics

All prokaryotic and eukaryotic cells secrete, in an evolutionary conserved way, extracellular vesicles (EVs), i.e., membrane-derived nano- and microvesicles [1]. For a long time, these vesicles were supposed to be either a waste removal system, products of cellular damage, or experimental artefacts [2]. Nowadays, EVs are recognized as specific cellular components performing different biological functions [3,4].

EVs are classified on the basis of the different sizes, cellular compartment of origin and localization, either inside or outside the cells [1]. Among them, exosomes, microparticles, shedding vesicles, apoptotic bodies, tolerosomes, prostasomes and prominosomes have been distinguished [5]. Two main processes for EV formation have been identified: some EVs, such as exosomes, apparently derive from exocytosis of multivesicular bodies, a part of the endosomal system including primary endocytic vesicles, early and late endosomes, and lysosomes [6]; otherwise, EVs may form from the direct budding of the cell membrane [2].

Exosomes, firstly identified by Johnestone in 1987 [7], represent a homogenous class of EVs in terms of size (30–150 nm), density (1.13–1.19 g/mL [8,9]) and membrane composition, differently from the other classes of EVs, which are characterized by higher heterogeneity [10,11]. As exosomes derive from the membrane of late endosomes [1], their proteome is particularly rich in tetraspanins (CD9, CD63, CD81 and CD82) and heat shock proteins (HSP70, HSP90), but also includes transmembrane proteins that are specific to the parent cell; for example, exosomes deriving from platelets contain P-selectin and intercellular adhesion molecule-1, while α- and β-chains of integrins are expressed on the membrane of exosomes deriving from dendritic cells, reticulocytes and T-cells [12]. As for exosome lipid composition, phosphatidylserine, sphingomyelin, cholesterol, ceramides, and ganglioside GM3 are particularly abundant [1]. Sharing the same biogenesis, exosomes and apoptotic bodies present analogous membrane topology, with the interior side of the vesicle corresponding to the cytosolic side of the parent cell membrane, although phosphatidylserine is specifically exposed on the outer leaflet of the vesicle membrane, due to the activity of enzymes such as flippase, floppase and scramblase [11]. Moreover, exosomes carry nucleic acids such as mRNA, siRNA, miRNA and DNA fragments.

### 1.2. Biological Function

By vehiculating proteins and genetic material, exosomes are involved in cell-to-cell communication by molecule transfer from donor to nearby, as well as distant, recipient cells [13,14]. Some authors have already reviewed the physiologic functions of exosomes in the immune system [15,16]; for instance, an in vitro study on both human and murine models evidenced that exosomes deriving from lymphocytes stimulate CD4+ T cell clones, revealing that these EVs might be involved in the transfer of peptidic signals among immune cells [8].

**Figure 1:**
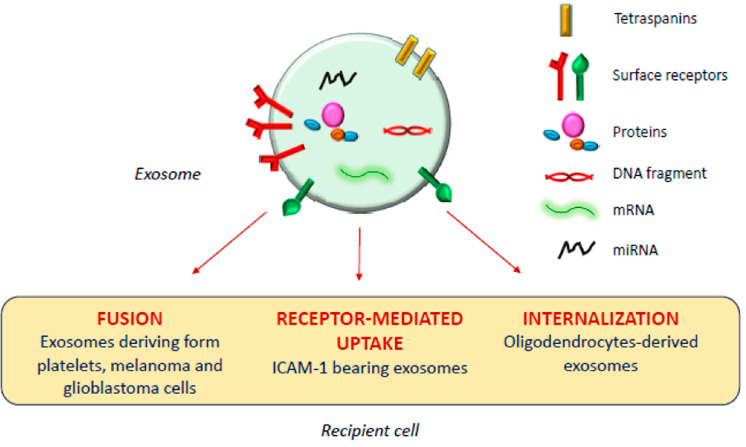
Exosomal mechanism of interaction with recipient cells.

The exosomal mechanism of interaction with recipient cells has not been clarified yet (Figure 1). Some studies reported that exosomes fuse with the plasma membrane of the recipient cell, releasing their content in the cytoplasm [17,18]; as observed by Parolini et al. [19], the fusion process might be facilitated under acidic pH, typical of the tumor intracellular environment.

Actually, besides shuttling bioactive molecules among healthy cells, exosomes also play a crucial role under pathologic conditions such as cancer, and neurodegenerative, cardiovascular, infectious and respiratory diseases [14,20,21,22,23].

Tumor cells release high amounts of EVs, and several studies report a possible involvement of tumor-derived exosomes in different phases of tumor formation and progression, as reviewed in [24], although opposing pieces of evidence have been reported by different research groups. Al-Nedawi et al. [25] observed that glioblastoma cell-derived EVs membranes are enriched in a mutant form of the epidermal growth factor receptor, which promotes anti-apoptotic pathways and increases the anchorage-independent growth capacity; on the other hand, Ristorcelli et al. [26] evidenced that EVs secreted by pancreatic tumor cells, due to their high membrane content of cholesterol and sphingomyelin, induce activation of the mitochondrial apoptotic pathway in tumor cells, and Zitvogel and his group [27] even demonstrated the capability of dendritic cell-deriving exosomes to inhibit tumor growth in vivo.

Besides membrane composition, the exosomal content may also influence tumor progression. Some studies evidenced the role of miRNA present in tumor-derived exosomes in promoting neovascularization and angiogenesis [28,29]; also, a recent work of Gerloff et al. [30] evidenced that cutaneous melanoma-derived exosomes are enriched in miR-125b-5p, which induces a tumor-promoting phenotype in tumor-associated macrophages, while Kurahashi et al. [31] observed increased miR-204-5p levels in urinary exosomes of transgenic mice used as a model of a rare form of renal cancer.

An important aspect of tumor progression is the capacity of cells to elude the immune system, and several studies demonstrated that tumor-derived exosomes are involved in this process. Chalmin et al. [32] isolated exosomes deriving from different murine and human cancer cell lines and identified the interaction between Hsp-72, associated with the exosome membranes, and Stat3, expressed by the parent cells, as the key factor inducing the immunosuppressing activity of both mouse and human myeloid-derived suppressor cells.

Moreover, several papers highlighted the involvement of tumor-derived exosomes in the metastastic process [33,34,35,36]. For example, Peinado et al. [37] demonstrated that the highly metastatic behavior of primary melanomas might be ascribed to the abundant generation of exosomes influencing bone marrow progenitors, although different pieces of evidence were obtained in a replication study in 2018 [38]. Ramteke et al. [39] demonstrated that prostate cancer cell-derived exosomes, secreted under the hypoxic conditions typical of the malignancy, enhance the invasiveness of the tumor through induction of the cleavage of E-cadherin, a protein involved in the adherens junctions among epithelial cells. Another study [40] demonstrated that exosomes deriving from bladder cancer cells promote lymphatic metastasis through the action of a long non-coding RNA vehiculated by the vesicles; similarly, miR-105, miR-122 and miR-200-containing EVs promote breast cancer cell metastasis [41,42,43] and miR-221-containing exosomes derived from gastric cancer mesenchymal stem cells were found to promote migration of human gastric cancer cells in vitro [44].

### 1.3. Applications in Therapy

Exosomes are endowed with several characteristics suitable for drug delivery: they are nano-sized, non-toxic, biocompatible, scarcely immunogenic, and possess targeting ability and organotropism [45]. Indeed, exosomes are similar to small unilamellar liposomes in terms of size and capacity to carry both hydrophilic and lipophilic molecules, but the asymmetrical lipid distribution and specific protein composition of exosome membranes justify their organotropism and homing ability [46], confirmed by the evidence that cancer-derived exosomes fuse preferentially with their parent cells [47].

However, the clinical translation of exosomes as drug carriers is affected by several technical issues, including low production yield, considerable structural heterogeneity and complexity, difficulties in drug loading and in developing standard, scalable, and cost-effective GMP procedures for exosome isolation and purification [48]. To overcome these issues, bioinspired exosome-like vesicles have emerged as an alternative to naturally derived exosomes. Most of the artificial exosome-mimetic systems proposed to date stem from liposomes—the so-called hybrid exosomes derive from the fusion of exosome and liposome membranes [49]—or are obtained by serial extrusion of a parent cell suspension through decreasing pore size membranes [50].

Many research groups have developed exosomes and exosome-mimetic systems as nanocarriers for cancer treatment [45], proposing different techniques for vesicle isolation and purification, drug loading and surface functionalization [51,52]. The drug loading methods, in particular, can be classified in two main classes, endogenous and exogenous loading [52]. Endogenous loading includes the genetic modification of the parent cells, to have them to express specific proteins or nucleic acids to be included in the released vesicles, or their simple incubation with the drug to be loaded; exogenous loading implicates the incorporation of the drug in exosomes previously isolated from cell culture media or body fluids (urine, blood, saliva, breast milk, etc.).

Various active principles have been loaded into vesicles developed for the treatment of different types of cancer, such as doxorubicin [53,54], paclitaxel [55], gemcitabine [56], but also aspirin [57], imperialine [58], several miRNA [59,60,61,62,63,64] and mRNA molecules [65], tumor necrosis factor-α [66] and recombinant methioninase [67].

### 1.4. Applications in Diagnosis

Beyond their possible use as therapeutic active carriers, exosomes can be employed in the diagnostic field with two different approaches. Passive diagnostic applications involve the use of naturally derived tumor exosomes as cancer diagnostic and prognostic biomarkers [68,69], since, differently from circulating cancer cells, their abundance in blood allows for easy detection in small volumes of frozen plasma or serum [70]. By analyzing the proteomic and genomic profile of these exosomes, including mRNA, miRNA and mitochondrial RNA, it is possible to determine the type of tumor and its stage [68,71,72,73,74]. As an example, Zong et al. [75] developed silicon quantum dots (Si-QD) decorated with a CD63 aptamer to bind CD63 expressed on exosomes isolated from human breast SKBR3 cancer cells [12], thus obtaining a nanoprobe for super-resolution microscopy suitable for trafficking studies in live cells and for the investigation of exosome role in cancer metastasis. Moreover, Chen et al. [76] developed an exosome-based system for super-resolution microscopy, demonstrating the possibility of simultaneous dual-color imaging by immunofluorescent labeling of CD63 and HER2 molecules expressed on SKBR3-derived exosomes.

On the other hand, some researchers proposed to exploit exosomes as an active diagnostic tool, by manipulating them with compounds or nanoparticles (NP) detectable using different imaging techniques, such as optical fluorescence, computed tomography (CT), positron emission tomography (PET), single photon emission computed tomography (SPECT), and magnetic resonance imaging (MRI), for their use in the diagnosis of some forms of cancer that are difficult to reach, such as brain tumors, or in the early detection of cancer recurrences and metastasis, within a precision medicine approach [77].

**Figure 2:**
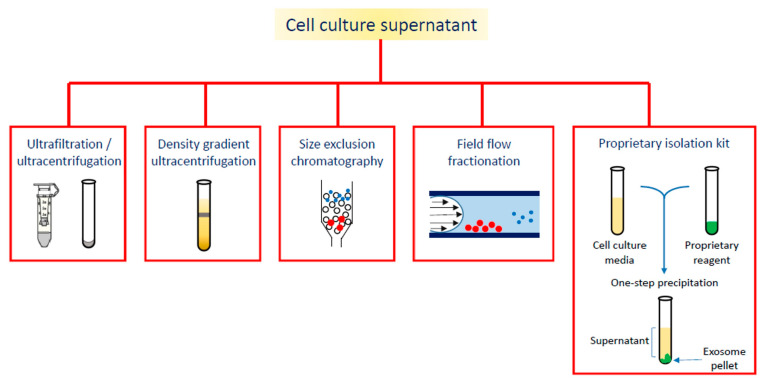
Different strategies for exosome isolation.

On the wave of growing interest in exosome and EVs applications, the aim of this review is to summarize the diagnostic or theranostic platforms based on exosomes (Table 1) or exosome-mimetic vesicles (Table 2) that have been developed so far, classified on the basis of the labeling probes; in particular, the review focuses on the diverse manufacturing (Figure 2), loading and surface modification procedures, and on applications in oncology; the final paragraph briefly reports on exosome applications in other pathologic conditions.

## 2. Nanoparticle-Loaded Exosomes in Oncology

### 2.1. Superparamagnetic Iron Oxide and Ultrasmall Superparamagnetic Iron Oxide Nanoparticles

In order to obtain Evs detectable through non-invasive techniques, such as MRI, some research groups conceived to employ iron oxide NP as contrast agents. SPION (superparamagnetic iron oxide nanoparticles) and USPION (ultra-small superparamagnetic iron oxide nanoparticles) are both composed of maghemite, γ-Fe_2_O_3_, or magnetite, Fe_3_O_4_ [97], but, generally, SPION feature a size range from 50 to 150 nm, while USPION are smaller than 50 nm.

Glioma-targeting exosomes for imaging and therapy were developed by Jia et al. [78], who, through electroporation, loaded the exosomes with SPION and curcumin, a polyphenolic compound endowed with inhibitory properties against tumor cell growth [98,99]. To overcome the blood–brain barrier permeation restriction, the authors conceived to associate the natural biocompatibility of the exosomes with appropriate surface functionalization; this led to neuropilin-1 (NRP-1)-targeting exosomes, useful as a theranostic platform, since NRP-1 is a transmembrane glycoprotein overexpressed by glioma cells and tumor vasculature [100]. The exosomes were isolated from mouse macrophage Raw264.7 cells by sequential centrifugation; nanoparticle tracking analysis (NTA) revealed their diameter (113 ± 4.2 nm) and zeta potential (−28.1 ± 3.6 mV), while Western blot analysis confirmed the presence of the exosomal marker membrane proteins CD63 and CD81. The authors obtained the best SPION:curcumin mass ratio (1:1) in loaded exosomes by optimizing the electroporation parameters and using specific mass ratios for curcumin:exosomes (3:1) and SPION:exosomes (1:10). The loaded exosomes were conjugated with an NRP-1 binding peptide through classic click chemistry between an alkyne group bonded to the phosphatidylethanolamine molecules exposed on the exosome membranes and the azido group of the peptide. Using an FITC (fluorescein isothiocyanate)-labeled peptide and NTA analysis, the authors estimated that each exosome was decorated with 52 peptide molecules and that 1000 µg of exosome was loaded with approx. 4000 µg of SPION and curcumin, which caused slight modifications in size and zeta potential (122.7 ± 6.5 nm and −24.1 ± 2.2 mV). The peptide conjugation on the exosomal surface was confirmed through various experimental approaches, one of which involved the incubation of 1,1′-dioctadecyl-3,3,3′,3′-tetramethylindocarbocyanine perchlorate (CM-DiI)-labeled exosomes with human glioma U251 cells, followed by observation of the fluorescence distribution and overlapping of CM-DiI and FITC signals through confocal laser scanning microscopy (CLSM). By flow cytometry, it was calculated that 94.88 ± 5.11% of the cells were FITC positive, and the presence of SPION inside the cells was confirmed by Prussian blue staining. Moreover, the presence of curcumin was verified by the co-localization of the red signal of CM-DiI labeled exosomes and the green signal of curcumin under CLSM. An in vivo test performed on BALB/c nude mice transplanted into the right striatum with U251 glioma cells revealed persistent fluorescence intensity in the tumor region of animals treated with targeted exosomes labeled with CM-DiI, while MRI showed a clear and distinguishable signal attributable to SPION. Finally, the authors reported that not only did SPION and curcumin not interfere with each other, but they also exerted a synergistic effect on glioma cells growth when magnetic fluid hyperthermia was induced by application of an external alternating magnetic field [101].

### 2.2. Quantum Dots

Gold-carbon quantum dots (GCD), prepared using a microwave-assisted method, were used for fluorescent labeling of cancer-derived exosomes [79]. GCD are characterized by weaker fluorescence intensity compared to organic dyes, but they are more photostable; moreover, GCD are more biocompatible than QD, which include toxic components such as Pb, Cd and Se, and can be obtained with less complicated synthetic paths. To produce an exosome-specific nanoprobe, GCD were functionalized with rabbit anti-HER2 antibodies, because of the high expression of HER2 on exosomes deriving from breast cancer cell lines such as SKBR3 [102]. So, via electrostatic interactions, the GCD surface was modified with polyethylenimine, and amide bonds between the amino groups of the polymer and the carboxylic groups of the anti-HER2 antibody were formed. The obtained nanoprobes were characterized for size (2 nm) and morphology through TEM. Finally, the anti-HER2-functionalized GCD were first blocked with albumin, to prevent non-specific interactions, and then incubated with the SKBR3-derived exosomes. The uptake of the labeled exosomes in the cytoplasmic compartment of HeLa cells was evidenced in vitro by fluorescence microscopy; moreover, after labeling the lysosomes with a specific red fluorescent dye, the authors evidenced the co-localization of the red fluorescent signal with the green signal deriving from the GCD-labeled exosomes, revealing that the exosomes had been uptaken into the lysosomal compartment.

By changing the composition of QD, it is possible to exploit different imaging techniques and associate a therapeutic activity, as was demonstrated by Cao et al. [80]. Vanadium carbide (V_2_C) QD, known photothermal agents, were prepared by a chemical exfoliation process from V_2_C nanosheets, followed by a hydrothermal treatment; V_2_C QD, which showed a hexagonal structure at high-resolution TEM, were decorated with TAT (transactivator of transcription) peptide, which enables nucleus uptake, through surface functionalization with 2-arm methoxypoly(ethylene glycol) amine and subsequent conjugation with the peptide; both steps exploiting EDC/NHS coupling. Arg-Gly-Asp (RGD)-modified exosomes were produced from MCF-7 breast cancer cells by introducing 1,2-distearoyl-sn-glycero-3-phosphoethanolamine-poly(ethylene glycol)-RGD (DSPE-PEG-RGD) in the parent cell culture medium and harvesting the vesicles after 2 days. The TAT-functionalized V_2_C QD were encapsulated in the RGD-decorated exosomes through electroporation, obtaining the system called V_2_C-TAT@Ex-RGD, and the success of the process was confirmed by UV-visible and FT-IR spectra. Using an FITC-labeled anti-RGD antibody, the number of RGD molecules in V_2_C-TAT@Ex-RGD was calculated to be approx. 3.7 × 10^3^ per exosome, while the concentration of TAT (1.36 µmol/mg V_2_C-TAT) was determined by detecting the number of amino groups by a ninhydrin method. Immunogold TEM images confirmed the integrity of exosome membranes after QD inclusion and RGD functionalization; dynamic light scattering (DLS) gave 71 nm as the mean diameter for V_2_C-TAT@Ex-RGD and −26 mV as the zeta potential. An in vivo study on MCF-7 tumor bearing BALB/c nude mice evidenced that the V_2_C-TAT@Ex-RGD nanostructures accumulated in the tumor site without disassembling before organ sequestration. By CLSM on MCF-7 cells previously labeled with the 3,3′-dioctadecyloxacarbocyanine perchlorate (DiO) green fluorescent dye, and then incubated with V_2_C-TAT@Ex-RGD labeled with DiI, the authors verified the efficiency of RGD targeting and TAT-mediated nucleus penetration. Besides its suitability for fluorescence and MRI imaging, the near infrared-II absorbance and the photostability of the developed system make it ideal for photoacoustic imaging, as confirmed in MCF-7 tumor-bearing mice injected with V_2_C-TAT@Ex-RGD. Also the photothermal efficacy of the system was confirmed in vitro on three cell lines (MCF-7, A549 and NHDF) and in vivo on tumor-bearing nude mice subjected to 1064 nm laser irradiation for 10 min.

### 2.3. Gold Nanoparticles

**Figure 3:**
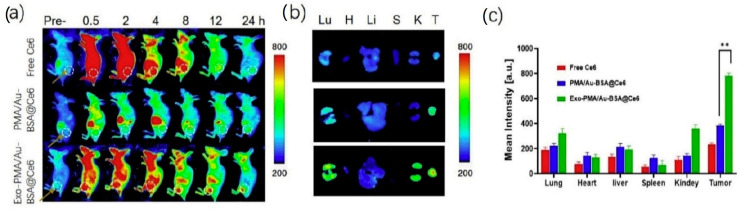
(**a**) Fluorescence imaging of mice bearing MGC-803 tumors intravenously injected with free Ce6, *PMA/Au-BSA@Ce*6 or *Exo-PMA/Au-BSA@Ce6* nanovehicles, obtained at the designated time points before and after injection in vivo. (**b**) Ex vivo fluorescence pictures of major organs (lung, heart, liver, spleen, and kidney) and tumors harvested from mice intravenously injected with samples of free Ce6, *PMA/Au-BSA@Ce6* or *Exo-PMA/Au-BSA@Ce6* nanovehicles after 24 h. The results are presented as mean ± SD (n = 5). (**c**) Quantitative biodistribution of free Ce6, PMA/Au-BSA@Ce6 or Exo-PMA/Au-BSA@Ce6 nanovehicles based on the average fluorescence intensity from major organs and tumor (n = 5). ** = *p* < 0.05 as specified in the Experimental section. Reproduced from [81].

Pan et al. [81] developed exosomes encapsulating gold NP for fluorescence imaging and photodynamic therapy of cancer. Gold NP were prepared by a two phase method [103], using the poly(isobutylene-alt-maleic anhydride) (PMA) amphiphilic polymer for phase transferring. Bovine serum albumin (BSA) was coupled to the PMA/Au NP through van der Waal’s forces, and then PMA/Au-BSA NP were incubated with increasing amounts of fluorescent chlorine-6 (Ce6), which is trapped by the BSA network. PMA/Au-BSA@Ce6 NP were embedded by electroporation into exosomes collected from the urine of gastric cancer patients by sequential centrifugations, yielding Exo-PMA/Au-BSA@Ce6 NP, whose efficiency of production and NP loading were confirmed by TEM images. Western blot analysis showed the presence of the proteic markers CD9, CD47, CD63 and CD81, confirming that the electroporation had not damaged the exosomes. Exo-PMA/Au-BSA@Ce6 were characterized by an average hydrodynamic diameter of 75 ± 7.6 nm and a zeta potential of −31.4 ± 4.1 mV. By CLSM and flow cytometry, the authors assessed the tumor-targeting ability of Exo-PMA/Au-BSA@Ce6 on human adenocarcinoma MGC-803 cells, evidencing 1.6-fold higher fluorescence intensity compared to the controls. Interestingly, the same experiment performed on Raw264.7 macrophages indicated low vesicle uptake, which might result in prolonged half-life in vivo. For the photodynamic therapy application, the authors observed a decrease in MGC-803 cell viability when treated with Exo-PMA/Au-BSA@Ce6 and irradiated at 633 nm. BALB/c-nude mice implanted with MGC-803 cells were used as a tumor model for in vivo biodistribution evaluations after tail injection of Exo-PMA/Au-BSA@Ce6 (Figure 3): a very high fluorescent signal was measured within the tumor tissue of Exo-PMA/Au-BSA@Ce6-treated mice after 24 h (2.1-fold higher than after 4 h). Finally, the therapeutic efficacy of the system was evaluated in vivo on MGC-803 tumor-bearing mice.

### 2.4. Polymeric Nanoparticles

Wu et al. [94], with the aim of developing an activatable biomimetic nanoprobe to enhance the target-to-background ratio in cancer detection, coated polymeric NP with cell membranes to achieve tumor homing. According to the authors, this strategy might overcome the actual limitations of cancer diagnosis and personalized therapy. Their idea was based on the synthesis of pH-responsive porous coordination polymer nanoparticles (PCP NP) encapsulating doxorubicin: at pH > 5.6, doxorubicin fluorescence was quenched because of the fluorescence resonance energy transfer with PCP NP, while, at pH < 5.6 (typical of lysosome/endosome content), PCP NP were degraded and doxorubicin fluorescence was activated, providing a cancer-localized high signal-to-noise ratio. PCP NP were prepared by adding FeCl_3_ aqueous solution to a mixture of bisphenol, dopamine hydrochloride, ammonium hydroxide solution and polyvinylpyrrolidone-K30 and were characterized for average pore diameter (approx. 2.9 nm) by BET (Brunauer–Emmett–Teller) analysis. An optimized amount of doxorubicin was encapsulated through the PCP NP pores by simple incubation, causing an increase in NP size (from 195 to 213 nm) and zeta potential (from −8.2 to +7.1 mV, due to the doxorubicin positive charge); moreover, after doxorubicin loading, the total pore volume and the surface area decreased. To coat the PCP NP with cell membranes, human hepatoma Bel-7402 cells were dispersed in a membrane protein extraction buffer and submitted to freeze–thaw cycles followed by sequential centrifugations. The isolated cell membranes were mixed with the doxorubicin-loaded PCP NP and extruded through a 400 nm polycarbonate porous membrane; after cell membrane coating, the nanoprobe size increased to 244 nm and the zeta potential became negative again, with a value of −15.4 mV. The vesicles were incubated with different cell lines, and CLSM and flow cytometry evidenced the successful homotypic targeting on Bel-7402 cells and a time-dependent increase in doxorubicin fluorescence intensity. Finally, the MTT assay confirmed the cytotoxic activity of the doxorubicin-loaded nanoprobe.

## 3. Transition Metal-Labeled Exosomes

Another effective strategy for Evs application as tumor diagnostic tools is labeling with metallic compounds detectable by MRI, PET, and SPECT.

In a proof of concept study, Abello et al. [85] developed a system for in vivo tracking of gadolinium (Gd)-labeled exosomes in osteosarcoma. Exosomes were isolated from human umbilical cord mesenchymal stem cells (HUC-MSC) by sequential centrifugations; their mean hydrodynamic diameter, measured by DLS, was 171 ± 42 nm (polydispersity index PDI 0.043 ± 0.03), while the zeta potential was −16.03 ± 0.72 mV. To label the exosomes, the authors synthesized a DSPE-DOTA (1,4,7,10-tetraaza cyclododecane-1,4,7,10-tetraacetic acid) conjugate that complexed Gd. Gd-DOTA-DSPE was included in the exosome membranes by the lipid insertion technique (1:1 ratio of exosomes:Gd-DOTA-DSPE) and the resulting Gd-labeled exosomes (Gd-Exo) showed an average diameter of 148 ± 3 nm (with an increased PDI of 0.36 ± 0.001); the successful insertion of Gd-DOTA-DSPE in the membranes was demonstrated by the zeta potential decrease (−19.70 ± 0.82 mV). As the presence of free Gd is a well-known limitation in the use of Gd-based contrast agents [104], in order to verify the absence of Gd leakage from the complex, the authors performed a Gd release study comparing Gd-Exo with Magnevist^®^ (gadopentetate dimeglumine), used as reference: Magnevist^®^ displayed a burst release (20%) of Gd in the first 4 h, while after 72 h Gd-Exo released only 2% of the ion. By CLSM the authors confirmed exosome internalization in mouse osteosarcoma K7M2 cells using Rhodamine B (RhB), and flow cytometry revealed that exosome internalization was time-dependent, reaching the maximum (40%) after 24 h. Gd-Exo and Gd/RhB double-labeled exosomes were investigated on K7M2 mouse and 14B human osteosarcoma cells: while the single and double-labeled exosomes showed the same dose-dependent antiproliferative effect on K7M2 cells, Gd-Exo caused no dose-dependent inhibition of 14B cell growth but, conversely, Gd/RhB exosomes induced a dose-dependent proliferative effect. The mechanism of proliferative inhibition has not been clarified, although induction of apoptosis was excluded. An in vivo test was carried out on immunodeficient NU/NU nude mice bearing K7M2 osteosarcoma, and MRI images evidenced a clear accumulation of the vesicles in the tumor area 90 min after injection. A biodistribution study performed by inductively coupled plasma-mass spectrometry (ICP-MS) highlighted that liver (38%), kidney (8%) and spleen (2%) were the organs involved in vesicles elimination. Importantly, the authors evidenced higher tumor accumulation of Gd-Exo (18%) compared to Magnevist^®^, explainable with the biological origin of this nanocarrier. The authors also compared the fluorescence bioimaging results of exosomes and NP labeled with DiR (1,1′-dioctadecyl-3,3,3′,3′-tetramethylindotricarbocyanine iodide), a near-infrared fluorescent cyanine dye, using the same animal model, and observed that the tumor fluorescence intensity from Exo-DiR increased more slowly but eventually doubled the one from NP-DiR. The authors concluded that HUC-MSC exosomes accumulated in mouse osteosarcoma tumors 24–48 h post-injection and that Gd labeling was more specific and accurate compared to fluorescence imaging.

**Figure 4:**
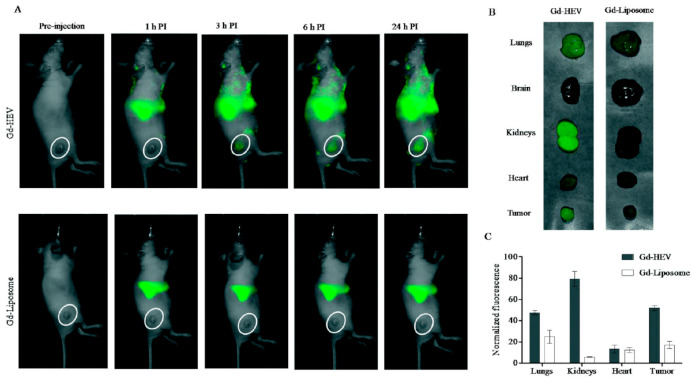
In vivo study by fluorescence bioimaging. (**A**) Time-dependent fluorescence images of mice injected with near-infrared DiR dye-labeled Gd-conjugated liposomes (Gd-HEVs) and Gd-liposomes, (**B**) bioaccumulation of Gd-HEVs and Gd-liposomes in organs of mice 24 h post-injection, and (**C**) quantification of fluorescence in organs harvested from Gd-HEV and Gd-liposome-treated mice. Fluorescence data were normalized by subtracting the fluorescence of the least fluorescent organ (brain) to picture the relative differences of fluorescence in different organs with respect to different particles. Reproduced from [95].

A strategy to maximize Gd delivery to the target site, prolonging the contrast agent circulation time and minimizing the dose to avoid toxic side effects, was proposed by Rayamajhi and his group [95]. These authors developed hybrid vesicles fusing macrophage-derived Evs with Gd-conjugated liposomes (Gd-HEVs) for MRI application. Evs were isolated from mouse macrophage J774A.1 cells by centrifugation, while liposomes composed of Gd-lipid, egg phosphatidylcholine and cholesterol (20:50:30) were prepared by the thin-film hydration method. The Gd-lipid amount was optimized by selecting the formulation with the best stability in terms of size, and the resulting Gd-liposomes were labeled with fluorescent RhB via hydrophobic insertion. The hybrid vesicles were obtained by mixing Gd-liposomes with the Evs in a 5:1 lipid:protein weight ratio and vortexing, followed by sonication and extrusion through a 200 nm polycarbonate porous filter. The Gd-HEV mean hydrodynamic diameter was 127 ± 2 nm (PDI 0.18 ± 0.01), and the zeta potential was −33 ± 4 mV; they featured good serum stability after 30 days with only a slight increment in size (up to 160 ± 10 nm) and PDI (up to 0.3 ± 0.05). Bradford assay, Fourier-transform infrared spectroscopy, sodium dodecyl sulfate–polyacrylamide gel electrophoresis and dot blot analysis confirmed that Gd-HEVs maintained the marker proteins (CD9, CD11b, CD63, CD81 and TSG101) of naive Evs; fluorescent-based energy transfer (FRET) analysis confirmed the success of the membrane fusion process. Gd^3+^ release measured through ICP-MS was less than 0.2% after 24 h, 1.5% at 48 h and 3.4% at 72 h, compared to Magnevist^®^, which released approx. 10%, 18% and 23% Gd^3+^ at the same time points. The safety of this contrast agent was also evaluated in vitro on mouse osteosarcoma K7M2 and normal fibroblast NIH/3T3 cells, resulting in over 70% cell viability at the highest concentration tested (0.2 mg/mL). The magnetic properties of Gd-HEVs were studied using 3T clinical MRI, highlighting a contrast enhancement as compared to Magnevist^®^ used at a similar concentration. In vitro tests on K7M2 and NIH/3T3 cells transfected with early endosome green fluorescent protein revealed a co-localization of the red RhB-labeled HEVs and green early endosome, especially in K7M2 cancer cells, as quantified by a CLSM imaging analysis program. By the same study, Gd-HEVs showed higher internalization than naïve Evs and Gd-liposomes in K7M2 cells. Gd-HEVs retained the typical functionality of Evs, but the authors were not able to explain the doubled cytokine stimulation functionality, absent in Gd-liposomes and Magnevist^®^. The biodistribution was studied by fluorescence bioimaging and MRI in NU/NU immunodeficient mice bearing osteosarcoma (Figure 4): fluorescence bioimaging revealed Gd-HEVs enhanced accumulation in lungs, kidney and tumor, while a real-time MR image, taken during injection, showed Gd-HEVs retention in the blood vasculature without extravasation, while Magnevist^®^ exhibited immediate extravasation. In accordance with the magnetic property characterization, Gd-HEVs showed enhanced contrast intensity compared to Magnevist^®^ also in vivo, although Gd-HEVs did not show a clear improvement of contrast enhancement in the tumor area compared to the surrounding tissue. The low response was confirmed by ICP-MS on collected organs, revealing that only 0.63% of the Gd injected dose accumulated in the tumor, which, however, was still significantly higher than Magnevist^®^ (0.1%). The authors hypothesized that this low tumor homing might be due to a prolonged blood retention.

Banerjee et al. [86] developed a platform based on radiolabeled small Evs (sEVs) for PET/MRI imaging. sEVs were isolated by sequential ultracentrifugation of human umbilical cord blood mononuclear cells cultured under hypoxic conditions, which makes the vesicles more bioactive than those secreted under normoxic conditions [105]. sEVs were characterized by DLS, NTA and TEM (average diameter of about 100 nm, and zeta potential of −34 mV), and the surface expression of the protein markers CD9, CD45 and CD63 was confirmed by flow cytometry. The DOTA maleimidoethylacetamide derivative was then conjugated to the free thiol groups on sEVs surfaces (880 ± 150 DOTA per sEV) for complexation of the PET emitter ^64^Cu^2+^, which was assayed through ICP-MS, resulting in 30% complexation efficiency; DLS revealed no statistical changes in size and zeta potential of sEV-DOTA-Cu in comparison with the original sEVs. In vivo stability was evaluated by intravenous injection in C57BL/6J mice: after 4 h, more than 95% of sEV-DOTA-Cu was present as a complex in the blood. A biodistribution study on mice treated with sEV-DOTA-Cu evidenced that after 3 h the radioactive signal was still significant, compared to the control mice receiving only DOTA-Cu, which showed almost no radioactivity already after 1 h. The highest radioactive signal was localized in the liver (around 30%), followed by lung, kidney, bowel, stomach and brain (0.4–0.5%). The authors stated that this surface labeling procedure was superior to previously applied strategies using ^68^Ga-NOTA (2,2′,2″-(1,4,7-triazacyclononane-1,4,7-triyl)triacetic acid), which induced vesicle aggregation, and to ^99m^Tc encapsulation, which caused an increase in the salivary gland activity due to ^99m^Tc leakage [96]. However, the authors did not exhaustively deal with aspects relating to MRI.

^64^Cu^2+^ was also employed by Shi et al. [87] to label exosomes from breast cancer cells. The vesicles were functionalized with reactive-amine NOTA and were pegylated to enhance half-life and tumor retention. The average hydrodynamic diameter of NOTA-exosome-PEG, measured by DLS, showed a dramatic difference between the elaboration by intensity or number distributions (226.6 ± 17.9 nm and 63.6 ± 10.0, respectively). NOTA-exosome-PEG exhibited a clear difference of zeta potential (−3.3 ± 3.2 mV) compared to neat exosomes (−33.4 ± 2.2 mV) and NOTA-exosomes (−26.6 ± 2.5 mV). After 24 h of storage, ^64^Cu-NOTA-exosomes-PEG featured significantly higher serum stability (95.7 ± 0.9%) than ^64^Cu-NOTA-exosomes (80.4 ± 1.3%), probably because, according to the authors’ opinion, the PEG chains hindered the competitive non-specific binding of serum proteins. In vivo, PET imaging was performed on 4T1 tumor-bearing mice to evaluate the biodistribution of ^64^Cu-NOTA-exosomes and ^64^Cu-NOTA-exosomes-PEG after intravenous injection: while ^64^Cu-NOTA-exosomes displayed robust hepatic clearance and very short blood circulation time, ^64^Cu-NOTA-exosomes-PEG exhibited opposite features. Moreover, ^64^Cu-NOTA-exosomes-PEG gradually accumulated in the tumor (2.7% ± 0.3 ID/g), with three-fold higher tumor uptake 24 h post-injection compared to ^64^Cu-NOTA-exosomes. The authors observed that the presence of PEG also enhanced tumor contrast, as highlighted by the tumor/muscle ratios (3.5 ± 1.1 at 1 h, 6.1 ± 1.1 at 4 h, 7.4 ± 0.5 at 24 h). These results were confirmed by histological studies performed with pegylated exosomes labeled with a fluorescent dye.

HER2-targeted exosomes radiolabeled with fac-[^99m^Tc(CO)_3_(H_2_O)_3_]^+^ synthon for SPECT tumor imaging were studied by Molavipordanjani et al. [88]. Targeted exosomes were obtained by transfecting parent human embryonic kidney HEK293T cells with a lentiviral vector to produce exosomes expressing on membranes DARPin G3, a ligand of the HER2 receptor [106]. Radiolabeling was obtained by incubating fac-[^99m^Tc(CO)_3_(H_2_O)_3_]^+^ synthon with the exosomes (RCP 96.5%): the labeled exosomes showed a slight size increase at DLS, compared to unlabeled ones (92.8 nm, PDI 0.309 vs. 76.5 nm, PDI 0.299) and resulted stable in saline for 24 h (RCP 96%). Using a gamma-counter, the binding to different cancer cell lines was investigated, evidencing that SKOV-3 cells, which are characterized by the highest HER2 receptor expression, displayed the highest affinity; the authors confirmed that the binding was dependent on DARPin G3-HER2 interaction by saturating SKOV-3 cells with trastuzumab, an anti-HER2 antibody. The biodistribution in normal BALB/c mice and SKOV-3 xenografted nude mice revealed that the highest portion of radioactivity was accumulated in the liver and kidneys, while tumor tissue radioactivity uptake was only 2.75 and 1.47% of injected radioactivity/g, measured 1 and 4 h post-injection, respectively. Pre-injection of SKOV-3 xenografted mice with trastuzumab caused the disappearance of the signal in the tumor area, corroborating the hypothesis that ^99m^Tc-exosomes acted through active targeting even in vivo.

**Figure 5:**
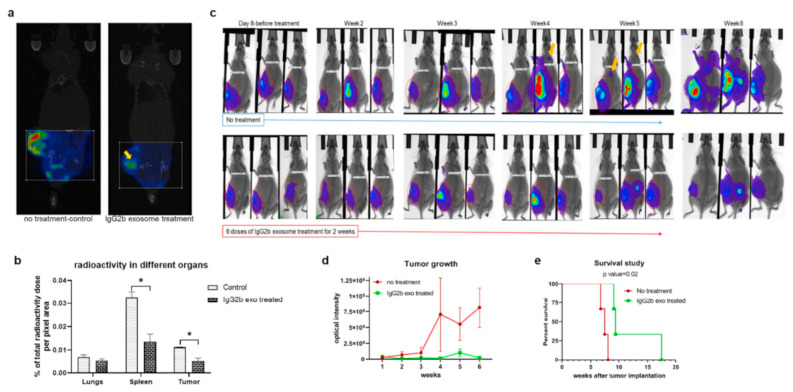
Treatment of 4T1 tumor-bearing animals with therapeutic engineered exosomes prevents tumor growth and metastasis and improves survival by depleting M2 macrophages. (**a**,**b**) Reconstructed and co-registered in vivo single photon emission computed tomography (SPECT)/CT images (coronal view) and quantification of subcutaneous syngeneic tumor-bearing animals (on the flank) injected with the ^99m^Tc-labeled precision peptide after 3 h. The group treated with therapeutic exosomes showed a lesser level of radioactivity in tumor (yellow arrow) and spleen compared to the untreated control group. Quantitative data are expressed in mean ± SEM, * *p* < 0.05; *n* = 3. (**c**) Optical images of 4T1 tumor-bearing animals treated with engineered therapeutic exosomes (lower panels) or without treatment (control), showing decreased tumor growth in treated animals compared to the control group. Metastatic foci in the control group were detected (yellow arrows) as early as at the fourth week from the injection, whereas no metastasis was detected in treated animals after 6 weeks. (**d**) Quantification of optical density of the tumor area also showed decreased tumor growth in treated group compared to control group. Quantitative data are expressed in mean ± SEm. *n* = 3. (**e**) Kaplan–Meier plot showing prolonged survival of the mice treated with therapeutic engineered exosomes. Log-rank test (Mantel–Cox) was applied to determine the significance of differences among the groups (*p* = 0.02). Reproduced from [89].

In the context of SPECT tracers, ^111^In-radiolabeled engineered exosomes were developed by Rashid et al. [89], with the aim of targeting pro-tumorigenic M2 macrophages, having a pivotal role in breast cancer cell dissemination. Exosomes were isolated by sequential centrifugations from human embryonic kidney HEK293 cells, transfected with a lentiviral vector to obtain vesicles expressing the CSPGAKVRC peptide, which specifically binds to CD206-positive M2 macrophages. Exosome hydrodynamic radius (92.2 ± 4.6 nm, measured by NTA) was not significantly different from the diameter of exosomes obtained from non-transfected cells (106 ± 14 nm). The binding ability of the engineered exosomes was tested in vitro on Raw264.7 macrophages treated with IL-3 and IL-4, stimulating CD206 expression. The engineered exosomes were labeled with DiI and binding and internalization were observed by fluorescence microscopy. To confirm the targeting ability in vivo, the same DiI-labeled exosomes were administered to 4T1 tumor-bearing Balb/c mice and tissue immunofluorescent staining confirmed the co-localization of exosomes and CD206-positive macrophages. The exosomes were labeled by incubation with In-111-oxine at room temperature for 30 min, following a previously described method [107]: the labeling efficiency was 98%, and, after 24 h of storage in FBS, more than 92% of ^111^In was still bound to the exosomes. In vivo SPECT and CT evidenced the accumulation of ^111^In-exosomes in tumor, lung (metastatic site), spleen, lymph nodes and bones of mice bearing 4T1 breast cancer (Figure 5). As a counterproof, a significant radioactivity reduction was observed in the tumors of a group of animals receiving Clophosome-A, a macrophage-depleting agent. A biodistribution study performed measuring the radioactive signal with a gamma-counter confirmed these results, revealing signals in kidneys and bladder, indicating that these organs were involved in the radiolabeled exosome excretion. Finally, the authors investigated the therapeutic potential of these targeted exosomes, further engineered to express the Fc portion of mouse IgG2, successfully inducing antibody-dependent cell-mediated cytotoxicity.

## 4. Other Systems as Potential Cancer Diagnostic Tools

### 4.1. Bioluminiscent Agent-Loaded Evs

Lai et al. [90] studied a bioluminescent method for the in vivo detection of Evs by exploiting the *Gaussia princeps* luciferase (Gluc). Luciferase reporters, differently from those conjugated to fluorescent proteins, do not require an excitation source for inducing their light emission; moreover, Gluc presents higher sensitivity compared to other luciferase-based reporters [108]. The researchers developed an engineered membrane-bound Gluc, by fusing it with both the transmembrane domain of a platelet-derived growth factor and a biotin acceptor peptide; then, using a lentivirus vector encoding for the engineered Gluc, they transfected human embryonic kidney HEK293-T cells, from which bioluminescent Evs were isolated by sequential centrifugation. In vivo tests on immunodeficient athymic mice injected with the Gluc-labeled Evs evidenced a prominent bioluminescent signal from the spleen. In a following study [91], the authors developed new reporter constructs for Evs membrane labeling by fusing the palmitoylation sequences (MLCCMRRTKQ) of a growth factor with either a green fluorescent protein (GFP) or tandem dimer Tomato (tdTomato), an orange fluorescent protein that, unlike GFP, does not require fixation for detection in histological tissue sections [109]. Plasmids encoding for palmGFP or palmtdTomato were inserted in a lentivirus vector, which was used to transfect HEK293-T cells, from which the Evs were isolated following a procedure similar to the one described in their previous work [90]. The researchers demonstrated that the palmGFP/palmtdTomato constructs could label the Evs of different sizes, with palmtdTomato resulting superior to the PKH67 fluorescent dye, which exhibited the tendency to aggregate, potentially leading to false-positive signals. Noteworthy is the application of this method to the CLSM observation of the dynamical exchange of Evs between palmtdTomato-293-T cells and palmGFP-glioblastoma cells. In an in vivo test performed on C57BL/6 mice implanted with mouse thymoma EL-4 cells expressing palmGFP, after 9 days, the presence of green fluorescent *punctae* was observed by multiphoton intravital microscopy, indicating the presence of different sized Evs.

### 4.2. Nanocluster-Loaded Exosomes

Several nanomaterials have been studied as fluorescent probes, and Tayyaba and his group [92] exploited the favorable properties of self-assembled Ag-nanoclusters and Fe_3_O_4_ NP to label exosomes that might be used for the diagnosis of cancer through fluorescence bioimaging. At the basis of this system is the observation that the low oxygen level in malignant cancer cells, due to a disproportion between their rapid growth and neoangiogenesis, induces the formation of fluorescent nanoclusters from some noble metal ions. Human hepatocellular carcinoma HepG2 cells were cultured in the presence of AgNO_3_ and FeCl_2_ with glutathione supplementation, to reduce the oxidative stress caused by the formation of the nanoclusters. The self-assembled nanoclusters biosynthesized by the cells were self-loaded into exosomes (as already demonstrated by other studies [110]), which were isolated through sequential centrifugations. TEM images demonstrated that no change in exosome morphology occurred after nanocluster loading, with a mean diameter of 50 nm, measured by DLS, and a zeta potential of +6.1 ± 0.9 mV. The cytotoxicity of the nanocluster-loaded exosomes was evaluated by MTT assay on both HepG2 and U87 glioblastoma cells, evidencing a fatal effect on both. The cargo-loaded exosome uptake was observed by CLSM, and flow cytometry confirmed their cancer cell targeting ability. From these pieces of evidence, the authors stated that the self-assembled nanocluster-loaded exosomes might be useful as cancer biomarkers and might also be applied for early cancer detection.

### 4.3. Metabolic Labeled Exosomes

Horgan et al. [93] developed a metabolic labeling strategy exploiting deuterium to study Evs using a confocal spontaneous Raman microspectroscopy system. Evs were isolated from MDA-MB-231 breast cancer cells, which were grown non-adherent in order to optimize Ev production. Deuterium was introduced into the cells by supplementing the cell culture medium with deuterium dioxide (D_2_O), or deuterated glucose (d-Gluc), or deuterated choline chloride (d-Chol). The authors observed that cells cultured in the presence of d-Gluc and d-Chol did not display significant variation in cell viability, which was slightly reduced in cells cultured in the presence of D_2_O. After Ev isolation, the authors used NTA to study the effects of the metabolic labeling on Evs size and concentration, and, via immunoblotting analysis, they verified the expression of three protein markers, CD9, CD63 and CD81. A small but statistically significant increase in Ev production was found in cells cultured in the presence of D_2_O, with no change in protein marker expression. By maintaining the labeled Evs under cell culture conditions for different periods, the researchers confirmed that the metabolic labeling remained stable for up to 4 h, while, after 8 h, a statistically significant reduction in the deuterium peak was observed, even if the signal remained detectable for up to 24 h. An in vitro test performed by Raman spectroscopic imaging on MDA-MB-231 cells incubated with D_2_O-labeled exosomes revealed the presence of deuterium throughout the cytoplasm, a proof that the Evs had not been degraded by the cells yet. Using a previously developed volumetric Raman imaging method [111], the authors also observed Ev distribution by 3D imaging. Finally, through an automated Raman spectroscopic image processing and analysis framework, they highlighted higher D_2_O-labeled Evs cellular uptake at 37 °C than at 4 °C, and reduced uptake for non-malignant breast epithelial cells (MCF10A) in comparison with malignant MDA-MB-231.

## 5. Exosomes Beyond Oncology

Although cancer diagnosis and therapy are the main application fields for exosomes and similar vesicles, a few different intervention areas have been explored in the latest years.

First, the exploitation of exosomes as diagnostic tools or drug carriers requires accurate preliminary investigation of their distribution pattern in vivo. Hwang et al. [96] conceived to label macrophage-derived exosome-mimetic vesicles with ^99m^Tc-hexamethylpropyleneamineoxime (HMPAO, also known as exametazime) for SPECT/CT tracing in living mice. ^99m^Tc-HMPAO is a suitable radiotracer for Evs labeling because, being highly lipophilic, it easily penetrates the lipid bilayers and is trapped inside the vesicle by reacting with the sulfhydryl groups of glutathione. Exosome-mimetic vesicles were prepared from murine macrophage Raw264.7 cells extruded through polycarbonate porous membrane filters, followed by purification through density gradient ultracentrifugation. Vesicle radiolabeling was carried out by first labeling HMPAO with ^99m^TcO_4_, followed by incubation of 185–370 MBq of ^99m^Tc-HMPAO with 100 μg/100 μL exosome-mimetic vesicles. Purification from free ^99m^Tc-HMPAO was performed by two different methods, either using a size exclusion column (RCP 99.6% ± 3.3) or by centrifugation (RCP 93.7% ± 5.4). No significant changes occurred in vesicles size (approx. 213 nm), measured by NTA before and after radiolabeling, and the presence of the exosome marker protein CD63 was confirmed. The serum radiochemical stability of ^99m^Tc-HMPAO vesicles was approx. 90% after 5 h. In vivo SPECT/CT imaging performed on male BALB/c mice showed radioactivity accumulation in the liver and spleen 30 min after injection, and in the salivary glands after 3 h, while no brain accumulation was evidenced. On the contrary, control mice treated with ^99m^Tc-HMPAO showed brain accumulation and delayed salivary gland uptake. Moreover, exosome-mimetic vesicles obtained from HB1.F3 human neural stem cells and labeled as described showed the same distribution as obtained with Raw264.7-derived vesicles. Finally, the in vivo biodistribution was investigated by ex vivo radioactivity counting at different time points, highlighting that the main difference in biodistribution between ^99m^Tc-HMPAO vesicles and ^99m^Tc-HMPAO pertained to brain and liver. The authors also evidenced the advantage of exosome mimetic vesicles over natural exosomes, as the amount of exosomes produced by Raw264.7 cells was half the amount of exosome-mimetic vesicles obtained by sequential extrusions of the parent cells, while the in vivo biodistribution was similar.

Busato et al. [82,83] developed an exosome-based diagnostic platform for neurodegenerative diseases by loading stem-cell-derived exosomes with commercial magnetite USPION. Murine adipose stem cells (ASCs), isolated from male C57BL/6 mice, were labeled with USPION following two different procedures: in the first one, a fixed number of ASCs were incubated with increasing concentrations of USPION for 24 and 72 h; in the second procedure, a fixed amount of USPION was incubated for 72 h with increasing cell number. To evaluate the internalization of USPION, the cells were stained by the Prussian blue method, and counterstained with nuclear fast red. Using a light microscope, the authors highlighted a dose- and time-dependent internalization of USPION in ASCs, and TEM analysis confirmed the success of the labeling procedure, with the USPION localized in the cytoplasmic compartment. After optimization of the MRI parameters, exosomes were isolated from ASCs incubated with USPION for 24 h, by using an exosome isolation kit based on targeted filtration. Through a bicinchoninic protein assay, exosome concentration was measured in terms of protein content. The quantification of internalized iron oxide NP in the exosomes was performed with a previously described procedure [112] based on the use of a potassium ferrocyanide solution and determination of the absorbance at 700 nm by UV spectrophotometry. After the in vitro MRI visualization of USPION-labeled exosomes immobilized in an agarose matrix, the authors performed an in vivo test on male C57BL/6 mice, which were intramuscularly injected with USPION-loaded exosomes and with plain USPION containing similar amounts of iron; the USPION-labeled exosomes were clearly detected by MRI and by histological analysis of gastrocnemius.

Betzer et al. [84] developed a non-invasive CT imaging platform based on exosomes labeled with gold NP (GNP) as a powerful diagnostic tool for various brain disorders. The exosomes, isolated from mesenchymal stem cells by sequential centrifugations, were analyzed by NTA for diameter (127.7 nm) and zeta potential (−29.2 mV). To optimize the size for exosome labeling, the authors prepared both 20 and 5-nm-sized GNP (TEM measured), following two different synthetic pathways. To prevent aggregation, PEG7 was used in both procedures and D-(β)-glucosamine hydrochloride was coupled to GNP to promote exosome internalization. The loading, carried out by incubation at 37 °C and confirmed by dark field microscope images, did not cause any significant change in exosome diameter, but induced a clear zeta potential decrease. Atomic absorption spectroscopy allowed the authors to observe a decrease in GNP uptake when the incubation was carried out at 4 °C. Thus, the authors hypothesized the involvement of an active energy-dependent process; in particular, they verified that the uptake was mediated by the GLUT-1 transporter by interaction with glucose moieties of the GNP coating, especially in the case of the 5 nm NP. Brain accumulation and whole-body distribution of the GNP-exosomes were studied after intravenous (IV) and intranasal administration (IN) in C57bl/6 male mice, by quantifying the gold amount in explanted organs: 1 h after administration, the amount of exosomes in the brain was significantly higher in the case of IN administration compared to the IV route, and 24 h after IN administration a substantial presence of exosomes was still detectable. Regarding whole-body distribution, the main difference between the two administration routes was found in the liver accumulation, which was higher for IV injection. In vivo CT images of a murine model of stroke showed that GNP-exosomes were detectable in the stroke area after 24 h, and this result was confirmed by region of interest and inductively coupled plasma (ICP) analysis. Finally, to establish whether the proposed exosome labeling protocol was a reliable and consistent imaging technique, the authors double-labeled the exosomes with GNP and red PKH26 dye and, comparing brain CT images with spectral unmixing fluorescence images, they were able to identify both exosomes and GNP in the same striatal slices, confirming that the nanoparticles were retained in the exosomes up to 24 h after labeling.

## 6. Conclusions

The unique properties of exosomes and the observation of the exosome-mediated cell-cell signaling imply that exosomes might be employed as nanocarriers for drug delivery, for both diagnostic and therapeutic purposes in oncology. Sequential ultracentrifugation is considered the gold standard procedure in exosome isolation, even if many research groups prefer the use of proprietary isolation kits based on selective exosome precipitation. Noteworthy is the modification of the parent cell culturing conditions applied by Banerjee et al. [86] in order to maximize Ev bioactivity.

Regarding the diagnostic application, MRI and CT-based methods seem to be the most promising techniques for Ev detection, and most research groups favor exogenous loading methods for the inclusion of the detectable agent. As for the therapeutic application, hyperthermia can easily be obtained by using SPION, GNP or QD as labels; few researchers have reported the application of exosomes for vehiculating drugs, perhaps due to the limited drug loading.

Despite the advances made in the last decades in the comprehension of the mechanisms by which exosomes are generated and about their functions, either in healthy or diseased conditions, the challenges in the development of efficient GMP protocols for their isolation and characterization are still to be solved. The Minimal Information for Studies of Extracellular Vesicles 2018 (MISEV2018) issued guidelines for nomenclature, parent cell collection and pre-processing, and Ev isolation, concentration and characterization [113]. However, a lack of uniformity in Ev size classification and characterization is still evident, and guidelines are still missing for Evs drug loading and functionalization.

Another important issue to be considered is the potential immunogenicity of exosomes and other Evs; in this review, we reported several studies employing murine cells as a source of extracellular vesicles, but, in the future, the use of human-derived Evs would be necessary for clinical application. Indeed, while some papers in the literature reported the absence of immunogenic effects of human Evs in mice, no data are available for the contrary.

Finally, deeper insights in the implications of using cancer-derived Evs are needed. In fact, by vehiculating proteins and genetic material deriving from tumor cells, the use of these vesicles as drug carriers might be a double-edge sword.

## Figures and Tables

**Table 1:** Theranostic platforms based on exosomes, classified by loading strategy.

Ref.	Labeling Strategy	Parent Cells	Exosome Isolation Method	Labeling Compound	Therapeutic Compound	Loading/Labeling Procedure	Surface Engineering	Detection Technique	Tests
[78]	Nanoparticle-loaded exosomes	Raw264.7 mouse macrophages	Sequential centrifugation	SPION	Curcumin	Exogenous (electroporation)	NRP-1 binding peptide by click chemistry	MRI	In vitro: U251 cells In vivo: BALB/c nude mice transplanted with U251 cells
[79]		SKBR3 breast cancer cells	Exosome isolation kit	Gold-carbon QD		Exogenous (incubation exploiting targeted loading through anti-HER2 antibodies)		Fluorescence imaging	In vitro: HeLa cells
[80]		MCF-7 breast cancer cells	Exosome isolation kit	Vanadium carbide QD		Exogenous (electroporation)	RGD peptide introduced by incubating exosomes with DSPE-PEG-RGD	Photoacoustic imaging	In vitro: MCF-7, A549, NHDF cells In vivo: tumor-bearing BALB/c nude mice
[81]		Urine of gastric cancer patients	Sequential centrifugation	Chlorine-6 labeled gold NP		Exogenous (electroporation)		Fluorescence imaging	In vitro: MGC-803, Raw264.7 cells In vivo: MGC-803 tumor-bearing BALB/c-nude mice
[82,83]		Murine adipose stem cells	Exosome isolation kit	USPION		Endogenous (cell incubation)		MRI	In vitro: exosomes immobilized in an agarose matrix In vivo: C57BL/6 mice
[84]		Mesenchymal stem cells	Sequential centrifugation	Gold NP		Exogenous (incubation)		CT	In vivo: C57bl/6 mice
[85]	Transition metal-labeled exosomes	Human umbilical cord mesenchymal stem cells	Sequential centrifugation	^68^Gd (complexed by DOTA)		Exogenous (lipid insertion technique with Gd-DOTA-DSPE)		MRI	In vitro: K7M2 mouse and 14B human osteosarcoma cells In vivo: immunodeficient NU/NU nude mice implanted with K7M2 cells
[86]		Human umbilical cord blood mononuclear cells	Sequential centrifugation	^64^Cu (complexed by DOTA)		Exogenous (reaction between the maleimide group of DOTA and thiol groups on exosome surface)		PET/MRI	In vitro: HUVEC In vivo: C57BL/6J mice
[87]		4T1 breast cancer cells	Sequential centrifugation	^64^Cu (complexed by NOTA)		Exogenous (reaction of NOTA with exosome surface proteins)	PEG decoration using PEG5k/NHS	PET	In vivo: 4T1 tumor-bearing BALB/c mice
[88]		Mouse macrophage HEK293T cells	Sequential centrifugation	^99m^Tc		Exogenous (incubation with *fac*-[^99m^Tc(CO)_3_(H_2_O)_3_]^+^)	DARPin G3 functionalization by transfection of the parent cells	Radioactive signal by gamma-counter	In vitro: SKOV-3, MCF-7, U87-MG, HT-29, A549 cells In vivo: BALB/c mice, SKOV-3 xenografted C57 nude mice
[89]		Human embryonic kidney HEK293 cells	Sequential centrifugation	^111^In		Exogenous (incubation with ^111^In -oxine)	CSPGAKVRC peptide, functionalized by transfection of the parent cells	CT/SPECT	In vitro: Raw264.7 cells In vivo: 4T1 tumor-bearing Balb/c mice
[90]	Bioluminescently labeled exosomes	Human embryonic kidney 293T cells	Sequential centrifugation	*Gaussia princeps* luciferase (Gluc)		Endogenous (transfection of the parent cells with a gene encoding for Gluc bound to a membrane protein)		IVIS imaging	In vivo: immunodeficient athymic nude mice
[91]		Human embryonic kidney 293T cells	Sequential centrifugation	GFP, tandem dimer Tomato		Endogenous (transfection of the parent cells with a gene encoding for palmGFP/palmtdTomato)		Multiphoton intravital microscopy	In vitro: 293T cells In vivo: C57BL6 (B6) mice implanted with mouse thymoma EL-4 cells
[92]	Nanocluster loaded exosomes	HepG2 human hepatocellular carcinoma	Sequential centrifugation	Ag-nanoclusters and Fe_3_O_4_ NP		Endogenous (parent cells cultured in the presence of AgNO_3_ and FeCl_2_ forming the nanoclusters)		Flurescence bioimaging, CT, MRI	In vitro: HepG2, U87 cells
[93]	Metabolic labeled exosomes	MDA-MB-231 breast cancer cells	Ultracentrifugation and size exclusion chromatography	Deuterium		Endogenous (parent cells cultured in presence of D_2_O/d-Gluc/d-Chol)		Raman spectroscopic imaging	In vitro: MDA-MB-231, MCF10A cells

**Table 2:** Theranostic platforms based on exosome-mimetic vesicles.

Ref.	Cell Line	Labeling Compound	Therapeutic Compound	Vesicle Preparation Method	Loading/Labeling Procedure	Detection Technique	Tests
[94]	Bel-7402 human hepatoma cancer cells	NP-encapsulated doxorubicin	NP-encapsulated doxorubicin	Coating of the NP with cell membranes through extrusion	Incubation	Fluorescence imaging	In vitro: Bel-7402, MCF-7, L-O2 cells
[95]	J774A.1 mouse macrophages	Gd-conjugated liposomes		Sonication and extrusion of the exosome/liposome mixture	Obtained during vesicle preparation procedure	MRI	In vitro: K7M2, NIH/3T3 cells In vivo: osteosarcoma—bearing NU/NU immunodeficient mice
[96]	Raw264.7 mouse macrophages, HB1.F3 human neural stem cells	^99m^Tc-HMPAO		Sequential extrusion of parent cells and density gradient centrifugation	Incubation	SPECT/CT	In vivo: BALB/c mice

## Abbreviations

ASCs: adipose stem cells; BSA: bovine serum albumin; Ce6: chlorine-6; CLSM: confocal laser scanning microscopy; CM-DiI: 1,1′-dioctadecyl-3,3,3′,3′-tetramethylindocarbocyanine perchlorate; CT: computed tomography; d-Chol: deuterated choline chloride; d-Gluc: deuterated glucose; DiI: 1,1′-dioctadecyl-3,3,3′,3′-tetramethylindocarbocyanine perchlorate; DiO: dioctadecyloxacarbocyanine perchlorate; DiR: 1,1′-dioctadecyl-3,3,3′,3′-tetramethylindo tricarbocyanine iodide; DLS: dynamic light scattering; DOTA: 1,4,7,10-tetraaza cyclododecane-1,4,7,10-tetraacetic acid; DSPE-PEG: 1,2-distearoyl-*sn*-glycero-3-phosphoethanolamine-poly(ethylene glycol); EV: extracellular vesicle; FBS: fetal bovine serum; FRET: fluorescent-based energy transfer; GCD: gold-carbon quantum dots; Gd-Exo: gadolinium-labeled exosomes; GFP: green fluorescent protein; Gluc: *Gaussia princeps* luciferase; GNP: gold nanoparticles; HEVs: hybrid extracellular vesicles; HMPAO: hexamethylpropyleneamineoxime; ICP: inductively coupled plasma; IN: intranasal; IV: intravenous; MISEV2018: minimal information for studies of extracellular vesicles 2018; MRI: magnetic resonance imaging; NOTA: 2,2′,2′′-(1,4,7-triazacyclononane-1,4,7-triyl)triacetic acid; NP: nanoparticle; NRP-1: neuropilin-1; NTA: nanoparticle tracking analysis; palm: fused with the palmitoylation sequence of a growth factor; PCP NP: porous coordination polymer nanoparticles; PDI: polydispersity index; PET: positron emission tomography; PMA: poly(isobutylene-alt-maleic anhydride); QD: quantum dots; RCP: radiochemical purity; RhB: rhodamine B; sEVs: small extracellular vesicles; Si-QD: silicon quantum dots; SPECT: single photon emission computed tomography; SPION: superparamagnetic iron oxide nanoparticles; tdTomato: tandem dimer Tomato; TEM: transmission electron microscopy; USPION: ultra-small superparamagnetic iron oxide nanoparticles.
